# Co-designing an intervention for cardiovascular disease risk assessment and management after hypertensive disorders of pregnancy in primary care

**DOI:** 10.1186/s12961-024-01269-6

**Published:** 2025-02-20

**Authors:** Kaylee Slater, Rachael Taylor, Karen McLaughlin, Craig E. Pennell, Karyn Forbes, Milena Marcetic, Clare E. Collins, Melinda Hutchesson

**Affiliations:** 1 School of Health Sciences, College of Health, Medicine and Wellbeing, University of Newcastle, Callaghan, NSW 2308 Australia; 2 Food and Nutrition Research Program, Hunter Medical Research Institute, Lot 1,Kookaburra Circuit, New Lambton Heights, NSW 2305 Australia; 3 School of Nursing and Midwifery, College of Health, Medicine and Wellbeing, University of Newcastle, Callaghan, NSW 2308 Australia; 4 School of Medicine and Public Health, College of Health, Medicine and Wellbeing, University of Newcastle, Callaghan, NSW 2308 Australia; 5 Kotara Family Practice, PO Box 256, Kotara, NSW 2289 Australia; 6 Australian Action On Preeclampsia, Royal Melbourne Hospital, PO Box 2144, Melbourne , VIC 3050 Australia; 7 School of Health Sciences, University of Sydney, Camperdown, Sydney, Australia

**Keywords:** Preeclampsia, Gestational hypertension, Primary care, General practice, Implementation science, Behaviour change wheel, Cardiovascular disease, Cardiovascular risk, Preventative health, Patient and public involvement

## Abstract

**Background:**

Women with a history of hypertensive disorders of pregnancy are at an increased risk of cardiovascular disease. Although clinical practice guidelines for management of hypertensive disorders of pregnancy recommend involvement of a general practitioner for ongoing cardiovascular disease preventative care, there are no intervention strategies embedded within primary care aimed at improving risk assessment or management for women after hypertensive disorders of pregnancy. The study aim was to co-design an intervention to improve implementation of cardiovascular disease risk assessment and management following hypertensive disorders of pregnancy for primary care settings in a local health district in New South Wales, Australia.

**Method:**

Using the Integrated Knowledge Translation framework, a series of five co-design meetings with the investigative team and end users were conducted online. Meetings were informed by the Behaviour Change Wheel framework for intervention development and incorporated research findings from a systematic review and meta-analysis, surveys and an online discussion. Data from activities and audio recordings following each meeting were analysed thematically using inductive–deductive thematic analysis. Results were summarized after each meeting, and findings used to inform ongoing intervention development.

**Results:**

The 18 end users included women with lived experience of hypertensive disorders of pregnancy (*n* = 8), obstetricians (*n* = 2), midwives (*n *= 5) and general practitioners (*n* = 3). Target priorities were to improve communication between hospital staff and general practitioners following the occurrence of hypertensive disorders of pregnancy and increase the knowledge of general practitioners and women regarding cardiovascular disease prevention after cardiometabolic pregnancy complications. Part 1 of the intervention is set within the hospital setting and delivered via physical resources to address the communication gap between hospital and primary care providers about the occurrence of hypertensive disorders of pregnancy. Part 2 is delivered via an update to an existing general practice education platform and through resources for use within consultations to provide education for women and general practitioners about cardiovascular disease prevention after hypertensive disorders of pregnancy.

**Conclusion:**

The Integrated Knowledge Translation and Behaviour Change Wheel frameworks aided in the development of a targeted intervention to improve implementation of cardiovascular risk assessment and management for women after hypertensive disorders of pregnancy, based on gaps identified in current primary care practice.

**Supplementary Information:**

The online version contains supplementary material available at 10.1186/s12961-024-01269-6.

## Introduction

Pregnancies complicated by hypertensive disorders of pregnancy (HDP) are more likely to result in poorer maternal and neonatal outcomes [1]. Additionally, there are long-term health impacts of HDP, which include a twofold to fourfold increased risk of developing cardiovascular disease (CVD) within 10 years postpartum, as well as future non-communicable and metabolic diseases, such as diabetes [2]. Internationally, clinical practice guidelines [3, 4] for management after HDP, recommend regular blood pressure monitoring by a general practitioner (GP), adoption of a healthy lifestyle, returning to pre-pregnancy weight within 12 months of giving birth and limiting inter-pregnancy weight gain. These guidelines also suggest that women should be informed of their increased risk of HDP re-occurrence and future health conditions [3, 4]. However, research suggests that GPs are not being made aware of women’s history of pregnancy complications, and there is a lack of continuity of care from hospitals to primary care, resulting in challenges for women to engage in CVD prevention in primary care [5, 6]. Interventions are needed to improve the implementation of clinical practice guidelines for management after HDP. A narrative review by Marschner et al. (2023) advised that interventions should be tailored to community needs, specifically suggesting that, to optimize peripartum and postpartum management of ongoing care, lifestyle-focused interventions should be co-designed with women and integrated within healthcare systems [7]. However, to date, there is a lack of research focused on developing interventions using a co-design approach to improve implementation of CVD risk assessment and management for women with a history of HDP in primary care.

Using a co-design approach to improving CVD care post-HDP allows researchers to obtain the perspective of health professionals working with the women, as well as that of the women themselves. Co-design in healthcare involves the partnership of individuals who work within the system, for example, healthcare staff (obstetricians, midwives and GPs), individuals who have lived experience of using the system (for example, women with a history of HDP) and the “designers” of the new system (for example, researchers designing interventions to improve health systems) [8]. Research emphasizes poor sustainability of outcomes from health service delivery and quality improvement interventions, as well as long-term implementation failure [8]. Therefore, co-design is essential for meeting the needs of specific health systems which vary nationally and internationally. Integrated Knowledge Translation (IKT) is a co-design methodology which refers to collaboration between researchers and decision-makers and/or stakeholders [9]. IKT engages end users, also known as knowledge users (those who are going to be using and/or benefiting), and includes a set of processes or phases that can result in the generation of knowledge to optimize healthcare delivery systems [9].

Therefore, in the current study, IKT principles were used to develop an intervention to improve the implementation of CVD risk assessment and management following HDP in a primary healthcare setting in Hunter New England Local Health District (HNELHD), New South Wales (NSW), Australia. The HNELHD is a health district within NSW that contains a major metropolitan centre, a mix of several large regional centres and many smaller rural and remote communities within its borders, thereby representing a diverse population [10]. The NSW Mothers and Babies report (2021) described that 13% of women in the HNELHD smoked during pregnancy (third highest behind the Far West and Murrumbidgee Local Health Districts), and that 51% of pregnant women were overweight and obese [11], risk factors for both HDP and CVD. Additionally, after giving birth, women in the HNELHD are discharged from hospital, and on-going care is shifted to a GP; therefore, HNELHD primary care was chosen as the setting to co-design a system- and population-appropriate intervention.

The implementation intervention was co-designed by following the Behaviour Change Wheel (BCW) capability, opportunity and motivation behaviour model (COM-B model) and the Theoretical Domains Framework (TDF). BCW is a framework designed to create a change in behaviour and provides a step-by-step guide to developing behaviour change interventions [12]. The BCW comprises a “behaviour system” at the hub, known as the COM-B model, encircled by intervention functions and then policy categories [12]. TDF is a validated theoretical framework covering a range of actions for the behaviour change of health professionals that are relevant for the implementation of best-practice recommendations [13].

Therefore, the aim is to describe how the BCW was used with IKT to co-design an intervention to improve implementation of CVD risk assessment and management following HDP in a primary care setting in the HNELHD in NSW.

## Methods

### Study design

**Fig. 1:**
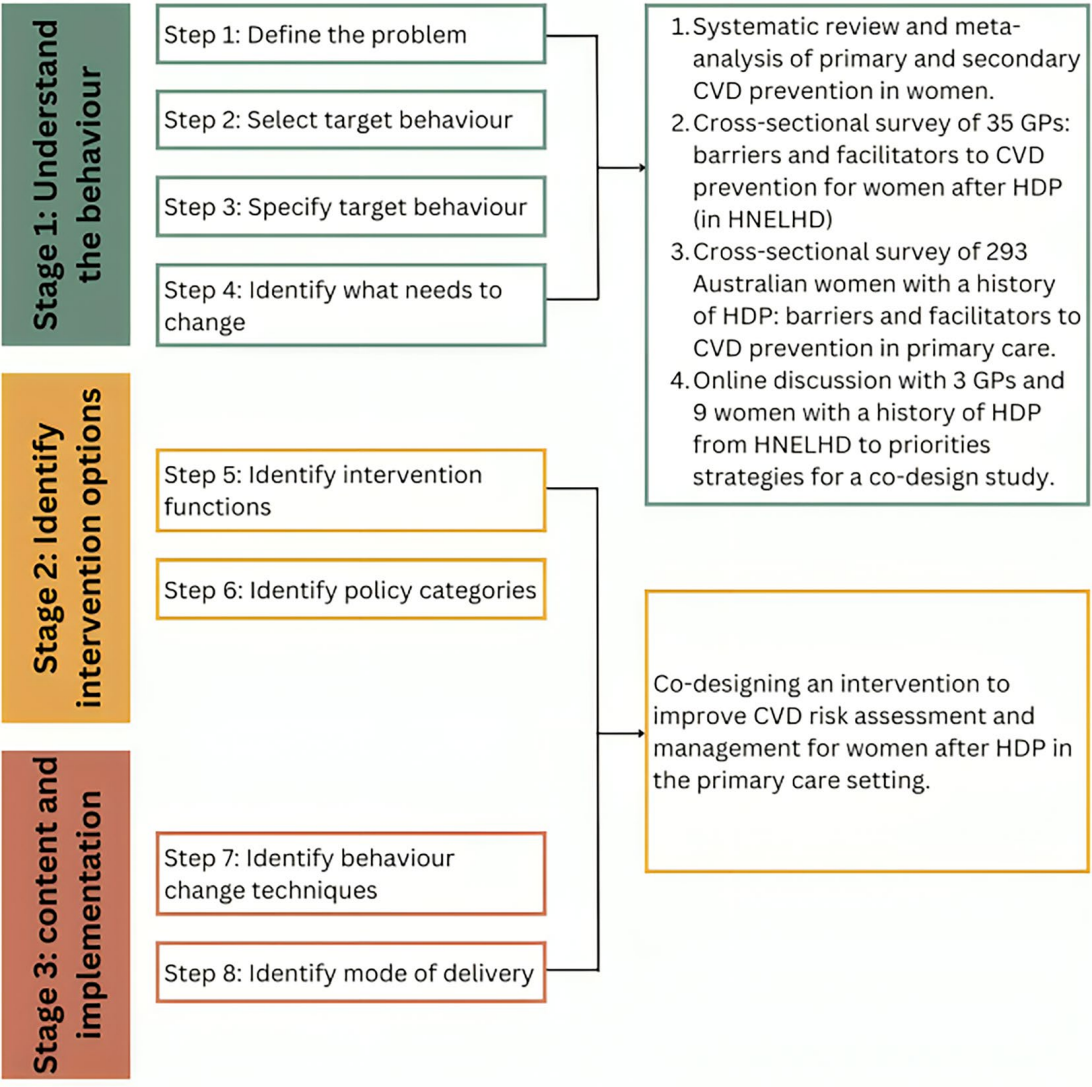
Stages and steps involved in intervention co-design, using the Behaviour Change Wheel by Michie et al. [12]

Figure 1 outlines the three stages and eight steps of the BCW framework used to co-design an intervention to improve implementation of CVD risk assessment and management following HDP for the primary care setting within the HNELHD. The BCW synthesizes behavioural strategies from 19 pre-existing models into a single framework, addressing the methodological limitations inherent in each individual model [12]. The BCW offers a systematic method for developing an evidence-based intervention, as well as the ability to tailor the intervention to meet the needs of women with a history of HDP.

In Stage 1 (understand the behaviour), findings from preliminary studies conducted by the investigative team were utilized to interpret effectiveness of CVD prevention interventions for women. This highlighted a lack of research within the primary care setting, and a need to understand whether health professionals, particularly GPs, were engaging in recommended CVD risk assessment and management with women following HDP in the HNELHD. Figure 1 demonstrates the steps undertaken and the studies conducted, and findings are presented in the Results.

Researchers also considered multiple international and national studies on CVD risk assessment and management after HDP from researchers in this field [14–20]. This demonstrated that women had not received recommended CVD care after HDP in their respective healthcare settings, which agreed with our study findings from Stage 1.

In Stage 2 (identify the intervention options), IKT was used to identify intervention options to include in the intervention design by selecting intervention functions and policy categories for each barrier identified in Stage 1. In Stage 3 (identify content and implementation options), behaviour change techniques (BCTs) were assigned to each intervention function/policy category, before deciding the mode of delivery. This study used the Behaviour Change Technique Taxonomy v1 (BCTTv1) developed by Michie et al. [21]. Stages 2 and 3 were completed through a co-design process, using IKT, whereby an investigative team and a team of end users were assembled. Ethics approval by the University of Newcastle Human Research Ethics Committee (H-2023–0153) was obtained for part of the study, and this manuscript was written in accordance with Guidance for Reporting Involvement of Patients and the Public (GRIPP-2) [22] reporting guidelines.

### >Participants in Stages 2 and 3 and study procedures

The investigative team (*n* = 8) included six researchers from a variety of health fields, including obstetrics and gynaecology, maternal dietetics and midwifery as well as two consumer members, a GP and a woman with lived experience of HDP. They were selected on the basis of their experience in clinical care in the HNELHD and/or research with women after HDP.

End users (midwives, GPs, obstetricians and individuals with lived HDP experience within the last 5 years) who lived or practised in the HNELHD were recruited primarily through snowballing and convenience sampling within the region. For health professional end users, members of the investigative team and health professionals who had already been recruited were sent an email invitation to share with other colleagues to promote the research study. Other recruitment methods for health professionals included a newsletter advertisement in the Australian College of Midwives monthly newsletter and sharing of the study on Twitter (now known as X). End users who were women with lived HDP experience were recruited through convenience sampling of those who had already participated in preliminary research (Fig. 1) and consented to being recontacted for participation in further research. In all, 38 prospective end users provided their written consent to participate (15 women with a history of HDP, 10 GPs, 9 midwives and 4 obstetricians). The final sample of end users included women with lived experience of HDP from 2017 onwards (*n* = 8), obstetricians (*n* = 2), midwives (*n* = 5) and GPs (*n* = 3). All end users were female and practising/living in metropolitan HNELHD (Greater Newcastle and Lower Hunter Valley).

Four online discussions took place via Zoom software (version 5.15.2, Zoom Video Communications, Inc. California, United States), and one final consultation with the investigative team took place via email from May 2023 to September 2023. The online discussions were led by K.S. and attended by R.T. and/or M.H. The investigative team consulted on three occasions (two via Zoom and one through email consultation), and the end users met via Zoom on two occasions to discuss and develop the intervention. Table 1 summarizes the focus and content presented at each meeting, and how it aligns to the BCW.

**Table 1:** A summary of the content presented at each meeting and pre- and post-meeting activities completed by researchers

	Participation	Focus and content	Pre- and/or post-meeting activity	Link to the Behaviour Change Wheel
Meeting 1 (investigative team)	Seven of the eight team members attended the meeting	Explaining how the study will proceed, presenting the findings from the preliminary research displayed in Fig. 1, and refining the structure of the co-design online discussions	Summarizing study methods and developing pre-reading materials for end users to view before their first meeting. Materials included a summary of preliminary research	Stage 1, Steps 1–4 (defining the problem, identifying the target behaviour, specifying the target behaviour and identifying what needs to change)
Meeting 2 (end users)	Held on two separate days, with 6 attendees on the first day and 12 attendees on the second	Using the APEASE criteria to brainstorm intervention ideas to address barriers to the provision of CVD prevention for women after HDP in primary care	Translated end users’ suggestions into 10 intervention functions and policy categories	Stage 2, Step 5 (identifying intervention functions) and Step 6 (identifying policy categories)
Meeting 3 (investigative team)	Four of the members (one obstetrician, midwife, GP and representative of women with HDP) completed the pre-meeting APEASE activity Seven of the eight team members attended the meeting	Using three of the APEASE criteria, the investigative team rated each of the 10 proposed intervention ideas, and then discussed and voted on their preferred intervention functions and policy categories	Calculated scores from the APEASE criteria and voting activity Commenced BCT coding of the proposed intervention Developed a translated summary of the intervention functions, policy categories and BCTs for end users	Finalized Stage 2, Step 5 (identifying intervention functions) and Step 6 (identifying policy categories)
Meeting 4 (end users)	Of the 18 end users, 17 completed a SWOT analysis of the proposed intervention ideas prior to the meeting A total of 12 end users attended the meeting (one obstetrician, two GPs, three midwives and 6 women with a history of HDP)	Appraisal of the proposed intervention via SWOT analysis (individual activity via online questionnaire facilitated on REDCap) and group discussion and voting on the delivery mode	Thematic analysis of the SWOT analyses Summarized version of the intervention, presented as each component of the BCW	Stage 3, Step 7 (BCTs) and Step 8 (mode of delivery)
Meeting 5 (investigative team)	Email consultation	Final feedback on the intervention plan via email, answering 2–3 questions: (1) does intervention parts 1 and 2 address the relevant barriers; (2) do you approve this intervention plan as a final version; and (3) if not, do you have any issues with the intervention plan, and if so, what would you change?	Summary of intervention plan, summary of study findings and manuscript preparation	

APEASE is a criterion that assists decision-makers to make context-based decisions during intervention design [12]. CVD, cardiovascular disease; HDP, hypertensive disorders of pregnancy; BCW, Behaviour Change Wheel; BCT, behaviour change techniques; APEASE, acceptability, practicality, effectiveness, affordability, safety/side-effects and equity; SWOT, strengths, weaknesses, opportunities, threats; REDCap, Research Electronic Data Capture

### Data collection and analysis

All completed tasks described in Table 1 were utilized as data. Additionally, each online meeting was audio and video recorded, and translated as summaries by K.S. Data collection, analysis and intervention development were iterative. Following each meeting, data were analysed and interpreted ahead of the next meeting and used to inform both the content of subsequent meetings and intervention development. Data from the strengths, weaknesses, opportunities and threats (SWOT) analysis (Meeting 4) were analysed via thematic analysis and were themed, and a deductive approach to analysis was taken, which involved data being coded according to pre-defined categories/techniques as part of the BCW process. As described in Table 1, further detail on how the content from each workshop was analysed, and how this informed ongoing intervention development, is included in the “pre- and/or post-meeting activity”.

## Results

Results are presented in line with the intervention development process described by Michie et al., with BCW steps presented sequentially, as per Fig. 1.

### Stage 1: understand the behaviour.

#### Step 1: define the problem

Our systematic review of primary and secondary CVD prevention interventions in women (Slater et al. 2022) revealed that lifestyle CVD prevention can reduce CVD risk markers, specifically systolic blood pressure and body mass index [23]. However, there is also a lack of research in CVD prevention for women after HDP, in healthcare settings and in younger populations (for example, in their childbearing years). Additionally, despite health professionals who care for women after pregnancy appearing to be aware of the link between HDP and CVD, it seems that the women themselves are largely unaware [24].

#### Step 2: select the target behaviour

Results from our survey of GPs suggest that they are unaware of guidelines relating to CVD prevention after HDP and are not specifically acknowledging obstetric history when undertaking CVD risk assessment [23]. This is reiterated through the women’s survey findings where the majority were unaware of their increased CVD risk [23, 25]. GPs reported their main barriers to providing recommended CVD care to women after HDP, which included lack of training in HDP and a lack of environmental support within their practices [23].

On the basis of findings from the two cross-sectional surveys, both GPs and women agreed on preferred strategies to improve CVD preventative care, which included increasing women’s awareness of increased CVD risk (*n* = 23, 70% and *n* = 62, 59% respectively) and improving communication between hospital and primary care [*n* = 22 (67%) and *n* = 58 (55%)] [23]. The priority strategies chosen in the online discussion with both these groups echoed survey findings.

Therefore, the collective decision was to primarily target CVD risk assessment and management following HDP in the primary care setting. We identified improving communication between hospital staff and GPs regarding occurrence of HDP and increasing knowledge of GPs and women regarding CVD prevention post-HDP as priority strategies to improve implementation of CVD risk assessment and management post-HDP.

#### Step 3: specify the target behaviour

In specifying the target behaviour using BCW, the targets would be:

- Who: GPs
- To whom: women with a history of HDP
- Where: primary care setting of the HNELHD
- What: improve patient discharge communication between hospital healthcare staff and GPs in primary care regarding the occurrence of HDP and increase knowledge of GPs and women of CVD prevention post-HDP.
- When/how often: immediate postpartum period initially and periodically thereafter based on clinical judgement by the participating GP.

#### Step 4: identifying what needs to change

Table 2 identifies what needs to change (that is, barriers to be addressed) to support GPs in providing CVD risk assessment and management to women after HDP using the BCW (including the COM-B model and TDF). This was determined by Stage 1 BCW and includes GP barriers to CVD risk assessment and management with women after HDP, the key areas for improvement categorized as COM-B classifications and barriers categorized by the TDF.

**Table 2:** Intervention components targeting provision of CVD risk assessment and management after HDP in primary care

Barriers to CVD risk assessment and management for women after HDP	COM-B	TDF
1. GPs are not being informed of their patients’ obstetric histories from hospital systems	Physical or social opportunity Indirectly influencing capability and motivation	Environmental context and resources
2. In GP practices, there are insufficient resources and training enabling them to assess and manage lifestyle risk factors among women following HDP	Psychological and physical capability Reflective and autonomic motivation	Environmental context and resources
3. GPs lack the confidence and skills to provide CVD risk assessment and management with women after HDP	Psychological and physical capability	Skills Emotions

CVD, cardiovascular disease; HDP, hypertensive disorders of pregnancy; COM-B, capability, opportunity and motivation behaviour model; TDF, Theoretical Domains Framework, GPs general practitioners.

### Stage 2: identifying intervention options

#### Step 5: intervention functions and step 6: policy categories

To address Barrier 1, end users deliberated over a range of intervention options including improvement of hospital discharge summaries, training of hospital staff on the importance of accurate data reporting in patient management software and improved coding of HDP in patient management software used in primary care. To address Barrier 2, end users deliberated over providing a range of different education options to the women themselves, such as antenatal education and advice on long-term CVD care following discharge from hospital. End users also suggested providing education and resources to GPs to use in consultation with postpartum women, and to share with the women post-consultation. To address Barriers 2 and 3, end users suggested providing updates to online information portals that GPs in the HNELHD have access to, as well as providing informal professional development and formal training. End user’s suggestions were translated into 10 intervention functions and policy categories by K.S. and reviewed by R.T. and M.H. (Additional File 1).

In Meeting 3 with the investigative team, the 10 proposed interventions were rated according to the APEASE criteria (Additional File 1), and the top 3 interventions were ranked (Additional File 2). This determined that education via communication/marketing and environmental restructuring via regulation would address Barrier 1, and education and enablement via communication/marketing and enablement via service provision would address Barriers 2 and 3.

### Stage 3: identifying content and implementation options

#### Step 7: behaviour change techniques and step 8: mode of delivery

BCTs were assigned to each intervention function by the investigative team. They were translated into three intervention ideas:

1. Develop a suite of resources on hypertensive pregnancies and heart disease risk for GPs to use in consultation with, and send to, women who had hypertensive pregnancies during their postnatal checks.
2. Arrange for Hunter New England HealthPathways postnatal check and hypertensive pregnancy modules to be updated to include information about heart disease prevention following hypertensive pregnancies. Health Pathways is an online clinical and referral information portal for health professionals to use in consultation with their patients.
3. Add information about hypertensive pregnancies and heart disease risk into the discharge summary bundle sent home with women for themselves and their GP.

Prior to Meeting 4, the 17 end users’ SWOT analyses of the three intervention ideas (Additional File 3), identified the common strengths for all three interventions to be accessibility and written and verbal education, and the common opportunities to be consistency of care and increased opportunity for education. Common weaknesses were reliance on health professional use and awareness of the intervention, and threats were reliance on patients to be able to effectively deliver the intervention and time constraints of health professionals.

To address the weaknesses and threats identified in the SWOT analysis, the discussion at Meeting 4 identified the following ideas:

1. Resources that outline a standardized model of care for GPs to follow.
2. Resources with reminders to schedule heart-health appointments, and timelines for follow-up, as well as links to further CVD prevention resources and resources with a video and visual component.

Modes of delivery included both physical and digital resources handed out within GP consultations and before discharge from hospital, and in-services and digital updates of available resources. These intervention delivery options were ranked by end users. Table 3 presents the most and least preferred ideas for each intervention.

**Table 3:** End users’ most and least preferred intervention delivery options for each of the three intervention ideas

Intervention idea	Most preferred options	Least preferred options
1	Resources with reminders for GPs and patients to schedule follow-up checks. Resources with a flow-chart for ongoing care and a one-page resource with a link/QR code to further information	Generic HDP card with a QR code to digital information Video information playing in the waiting room of GPs offices A large platform with a variety of generic CVD resources
2	HealthPathways update outlining a standardized model of care for GPs to follow with a link to the resource(s) developed in Intervention idea 1	Adding a blood pressure check reminder to the HealthPathways Links to lifestyle CVD prevention resources
3	Physical copy of the resource developed for Intervention idea 1 added to discharge summaries Including a prompt for follow-up in the Blue Book	Health professionals adding HDP information into an app Hospital staff including more personalized information in discharge summaries

HealthPathways is an online clinical and referral information portal for health professionals to use in consultation with their patients. The Blue Book is a newborn’s personal health record, which records a child’s health, illnesses, injuries, growth, development and vaccinations from ages 1 to 4 years. Parents or carers receive a free copy of the Blue Book when a child is born in NSW. CVD, cardiovascular disease; HDP, hypertensive disorders of pregnancy; GPs, general practitioners.

This was translated into mode of delivery by the investigative team, led by K.S., before presenting the finalized intervention plan, as components of the BCW. All investigative team members approved the final intervention plan as presented in Table 4.

**Table 4:** Intervention components targeting the provision of CVD management with women after HDP in primary care

Intervention part, barrier addressed	Intervention function	Policy category	Behaviour change techniques (BCTS)	Mode of delivery	Translation of behaviour change wheel components within the intervention
Intervention part 1 Addresses Barrier 1 (Table 2)	Environmental restructuring Change to discharge summary	Regulation Establish updated practice regarding the discharge summary	12.1 Restructuring the physical environment 7.1 Prompts/cues	In-service delivered to antenatal and/or nursing unit managers	One-page generic HDP resource added into both women’s discharge summary and Blue Book to be seen/read by GPs and women (BCT 12.1) HDP awareness sticker stuck onto the front cover of the baby’s Blue Book as an additional prompt for HDP follow-up in primary care (BCT 7.1)
	Education Informing and alerting women after HDP about long-term health risks and advice for follow-up	Communication, marketing Creation of a one-page physical fact sheet	5.1 Information about health consequences 7.1 Prompts/cues	Physical resources delivered by hospital staff in discharge pack	One-page generic HDP resource to provide information about ongoing CVD-risks after HDP (BCT 5.1) One-page generic HDP resource to provide information on recommended follow-up with a GP after HDP, including a flow-chart follow-up schedule and reminder to set-up ongoing heart health checks with GP (BCT 7.1)
Intervention Part 2a Addresses Barriers 2 and 3 (Table 2)	Enablement Revise content of the Hypertensive Pregnancy and Postnatal modules in HealthPathways	Service provision Establish services within HealthPathways to ensure adequate resources and information for all GPs	1.4 Action planning 4.1 Instruction on how to perform behaviour 12.1 Restructuring the physical environment	Digital notification of HealthPathways update	The Hypertensive Pregnancy module update (BCT 12.1) to include: • A section on long-term care, which will include recommendations from updated SOMANZ 2023 guidelines on CVD prevention after HDP (BCT 4.1) • Standardized model of care flow-chart for all women with a history of HDP, including recommended time points for ongoing heart health checks (BCT 1.4) • Update to also include a link to a digital copy of the one-page resource explained in Parts 1 and 2b Postnatal module update (BCT 12.1) will include: information suggesting all women should be asked about pregnancy complications during a postnatal check, with a link to the Hypertensive Pregnancy module (BCT 4.1)
Intervention Part 2b Addresses Barriers 2 and 3 (Table 2)	Education and enablement Providing resources to GPs for use with women after HDP	Communication, marketing Fact sheet for use within consultations and for GPs to send to women post-consultation	5.1 Information about health consequences 7.1 Prompts/cues	Resource delivered (digitally or physically) to women during face-to-face consultation with GP	As per Part 1, this resource will include information about CVD risks post-HDP (BCT 5.1), a recommended HDP flow-chart follow-up schedule and reminder for women and GPs to set up ongoing heart health checks, including risk factors to be assessed (BCT 7.1)

CVD, cardiovascular disease; HDP, hypertensive disorders of pregnancy; GP, general practitioner; BCT, behaviour change technique; SOMANZ, Society of Obstetric Medicine Australian and New Zealand

Therefore, the BCTs and mode of delivery identified for the intervention were those that all groups considered most appropriate to improve implementation of CVD risk assessment and management after HDP in the primary care setting. In summary, the proposed intervention has three parts. Part 1 would be delivered within the hospital setting using physical resources to close the communication gap between hospital and primary care of HDP occurrence and empower women post-HDP to be aware of monitoring for ongoing CVD risk. Part 2 includes 2a, to be delivered via an update to an existing GP education platform enabling improved provision of care, and Part 2b, delivered by resources for use within GP consultations.

## Discussion

This study describes a detailed explanation regarding use of IKT and BCW components within an implementation intervention to be embedded within the HNELHD primary care sector, aimed at changing health provider behaviour. Results from this co-design study indicate that an intervention aimed at improving implementation of CVD risk assessment and management for women after HDP should include strategies to improve communication of pregnancy complications during hospital discharge, communication to primary care and education for both women and GPs on CVD risk assessment and management after HDP. This study makes an important contribution to this field, particularly given that previous research has not used co-design nor developed an intervention for embedding within primary care.

Few research studies have sought to understand preferred CVD care for women after HDP. For example, an Australian qualitative study of 13 women with a history of HDP suggested that, when being transferred to primary care, women wanted to be informed about long-term medical follow-up [26]. A similar cross-sectional study of health professionals suggested that postpartum communication from the hospital system to community health was lacking [5]. Roth et al. also reported that health professionals wanted to improve health literacy amongst women after HDP, specifically requesting suitable and supportive materials to use with the women [5]. The intervention designed in this co-design study mirrored findings from other research, where health professionals and women with a history of HDP established a mechanism to address the limited communication between hospitals and primary care and suggested specific resources to be used within the primary care setting to educate women on their long-term health risks.

To date there have been few interventions specifically designed to reduce CVD risk factors in women with a history of HDP. In 2019, Lui et al. published a systematic review of randomized controlled trials that focused on lifestyle CVD prevention after HDP [27]. Lui et al. identified two published and four additional ongoing studies [27]. Whilst all those published acknowledge promising results, especially from online lifestyle interventions, they have not evaluated implementation of CVD prevention within their respective national health systems [18, 20]. Additionally, no studies included in the review have published details about the theoretical frameworks used to develop interventions [27]. The current intervention has been designed to be embedded within the primary care setting, and clearly recognizes the GPs role in long-term management of CVD risks for these women. We believe that this is the first time the BCW and co-design have been applied to the context of intervention development for CVD prevention for women with a history of HDP.

There are many benefits to using co-design and BCW in healthcare research. For example, co-design gives end users the opportunity to highlight their experiences and expertise, and meaningfully contribute to interventions addressing their needs [8]. Women with a history of HDP in this study were able to emphasize their lack of follow-up after pregnancy complications, and health professionals were able to discuss the gaps within the system, resulting in a better understanding of the complexities in the healthcare system. While co-design encourages greater equality in the relationship between end users and system designers, there are not many set procedures when using this approach to design an intervention. Therefore, this co-design study was guided by BCW, which resulted in an intervention design that can be described using defined terminology. The process also guided development of defined progress and milestones achieved after each meeting. Using BCW also encourages intervention designers to consider all intervention options and select those that are most likely to result in the desired outcome [12].

### Strengths and limitations

This novel study strengthens the theoretical foundations on which to develop an implementation intervention within the primary care setting, and for women with a history of pregnancy complications. Using co-design in this context incorporated the end users’ subjective interpretation of their experiences within the current health system to ensure the knowledge generated by this research can be embedded within real-world health settings. However, a limitation is that all end users were located within metropolitan areas of the HNELHD. Participants were encouraged to consider the broad geographical location of this health district, although their suggestions may have been more targeted to regional and/or metropolitan areas. A further strength is that the methods used to understand current behaviour within the context of post-HDP CVD care are explicit and transparent, allowing others to see how data from our previous systematic review [28] and surveys [23, 25] were mapped to COM-B and TDF, and used to iteratively develop the intervention plan. This study also captured both qualitative data from discussions within the co-design meetings and quantitative data via written feedback (Additional Files 1–3) prior to meetings, thereby providing participants with an opportunity to offer an unbiased opinion before attending the meetings. There are levels of consumer engagement in research, ranging from informing (provide information and awareness) to consumer-led (lead major activities) [29], and end users in this research were involved in providing information and perspective to design an intervention, but unable to be involved in every step of the BCW, such as assigning BCTs. However, there are various frameworks for co-design research [8], and researchers will pilot the implementation intervention to allow for further feedback and/or refinement.

### Implications for policy and practice

The intervention designed in this co-design study aims to improve health services targeting cardiovascular care for women after HDP by enabling team-based care, enhancing communication across health sectors and empowering GPs with updated resources. Targeted discharge summaries and updated in-clinic resources will ensure that primary care providers are well-informed about patients’ obstetric histories, enabling coordinated and continuous care. This will allow GPs to deliver comprehensive cardiovascular care after HDP, facilitating early detection and effective management of CVD. The intervention developed in this study should be pilot tested for feasibility and preliminary efficacy across the HNELHD in the next steps for this research, where modifications will be made to suit rural and remote community context, as well as content and delivery as needed. Additionally, findings from this study can inform future policy by demonstrating the importance of integrated care pathways and can identify effective strategies for postpartum CVD prevention, guiding resource allocation and health-system investment in impactful interventions.

## Conclusions

The current study describes the use of IKT and the BCW COM-B framework to address barriers to the provision of CVD care after HDP in primary care. We co-designed a three-pronged intervention to target the gap in communication between hospitals and primary care, as well as enable education for women and GPs on CVD prevention after HDP. This process was important for informing the design and intervention components that will be embedded within the primary care setting, prior to conducting a pilot feasibility and preliminary efficacy trial. Therefore, to bridge the communication gap that exists between hospitals and primary care, Part 1 of the co-designed intervention is implemented in a hospital setting and provided through tangible resources. To educate women and GPs on CVD prevention following HDP, Part 2 is provided through an upgrade to an already-existing GP education platform and tools for use within GP consultations. The systematic approach for this intervention development also has potential to inform intervention design in other populations and different health settings.

## Supplementary Information

Additional file 1.

Additional file 2.

Additional file 3.

## Data Availability

The original contributions presented in the study are included in the article or supplementary material. Further inquiries can be directed to the corresponding author.

## Abbreviations

HDP: Hypertensive disorders of pregnancy
CVD: Cardiovascular disease
GP: General practitioner
SOMANZ: Society of Obstetric Medicine Australia and New Zealand
ISSHP: International Society for the Study of Hypertension in Pregnancy
BCW: Behaviour Change Wheel
COM-B: Capability, opportunity and motivation behaviour model
IKT: Integrated Knowledge Translation
TDF: Theoretical Domains Framework
HNELHD: Hunter New England Local Health District
GRIPP-2: Guidance for Reporting Involvement of Patients and the Public
APEASE: Acceptability, practicality, effectiveness, affordability, safety/side-effects and equity
