# Supplementary material for: Co-designing an intervention for cardiovascular disease risk assessment and management after hypertensive disorders of pregnancy in primary care
Source: Health Res Policy Syst. 2025 Feb 20;23:23. doi: 10.1186/s12961-024-01269-6 (PMC11844034; doi:10.1186/s12961-024-01269-6)
Supplement: Supplementary file 1 — Additional file 1. [file 12961_2024_1269_MOESM1_ESM.docx]

# Additional File 1. Research team results from APEASE Criteria by intervention idea

| **Intervention idea** | **Intervention function** | **Policy category** | **Investigative team member** | **Acceptable (Y/N)**  Is the intervention likely to be liked or engaged with? | **Practical (Y/N)**  Is the intervention likely to be delivered and planned at the scale intended? | **Affordable (Y/N)**  Is the intervention likely to be implemented within a reasonable budget? |
| --- | --- | --- | --- | --- | --- | --- |
| **1** | **Training**  Education and training to hospital staff on the importance of accurate data recording in e-maternity, filling out discharge summaries, coordinating referrals to GP | **Service Provision**  Delivering in-person training programs, online webinars, pre-recorded trainings. | **1** | Y | N | N |
|  |  |  | **2** | Y | N | N |
|  |  |  | **3** | Y | Y | Y |
|  |  |  | **4** | Y | Y | Y |
| **2** | **Environmental restructuring**  Improvements to the discharge summary e.g., an additional page with HDP information on diagnosis, treatments given and suggested follow-up, adding an a generic HDP leaflet, automatic discharge summary to GPs | **Regulation and guidelines**  Establishing behaviours of practice regarding discharge summaries and documents that mandate practice, including the materials to include in discharge summaries | **1** | N | N | N |
|  |  |  | **2** | Y | N | N |
|  |  |  | **3** | Y | Y | Y |
|  |  |  | **4** | Y | Y | Y |
| **3** | **Enablement**  Appoint a CVD educator within the hospital that can facilitate education for women with HDP and coordinate discharge and postpartum care | **Service provision**  Establishing additional support services within hospitals to coordinate care | **1** | N | N | N |
|  |  |  | **2** | Y | N | N |
|  |  |  | **3** | Y | Y | N |
|  |  |  | **4** | N | N | N |
| **4** | **Education**  Antenatal education for women post diagnosis but before birth (community classes or on the wards) about follow-up after pregnancy complications. | **Service provision**  Delivering training sessions/programs to women in the antenatal period | **1** | N | N | N |
|  |  |  | **2** | Y | N | N |
|  |  |  | **3** | Y | N | N |
|  |  |  | **4** | Y | Y | Y |
| **5** | **Education**  Providing women with information on HDP, long-term risks and follow-up added to the Blue Book/given on wards | **Communication/marketing**  Creating brochures (paper or digital) to add to the bluebook/be handed out by midwives/specialists on HDP and recommended ongoing care | **1** | Y | Y | Y |
|  |  |  | **2** | Y | Y | Y |
|  |  |  | **3** | Y | N | Y |
|  |  |  | **4** | Y | Y | Y |
| **6** | **Environmental restructuring**  Encourage/audit coding of HDP diagnosis in GP clinical system | **Regulation**  Establishing this quality improvement activity within GP practices | **1** | Y | Y | Y |
|  |  |  | **2** | N | N | N |
|  |  |  | **3** | Y | N | Y |
|  |  |  | **4** | N | N | N |
| **7** | **Enablement**  Revise the antenatal and postnatal modules in Health Pathways (an online clinical and referral information portal for health professionals) | **Service provision**  Establishing services within Health Pathways to ensure adequate resources for all GPs | **1** | Y | Y | Y |
|  |  |  | **2** | Y | Y | Y |
|  |  |  | **3** | Y | Y | Y |
|  |  |  | **4** | Y | Y | Y |
| **8** | **Enablement and Education**  Providing information and resources for GPs to use in postnatal checks with women after pregnancy, including flow-charts, pathways for CVD assessment, lifestyle CVD interventions. | **Communication/marketing**  Creating templates for postnatal checks, flow-charts aligned with baby's milestones, resource suite for GPs to provide to patients and use within consultations | **1** | Y | Y | Y |
|  |  |  | **2** | Y | Y | Y |
|  |  |  | **3** | Y | Y | N |
|  |  |  | **4** | N | N | N |
| **9** | **Training**  Provide formal training for general practice nursing staff to enable them to facilitate postnatal education for women who have had HDP | **Service Provision**  Delivering in-person training programs, online webinars, pre-recorded trainings | **1** | Y | Y | Y |
|  |  |  | **2** | Y | N | N |
|  |  |  | **3** | N | N | N |
|  |  |  | **4** | N | N | N |
| **10** | **Training**  For GPs e.g., universal basic education for GPs providing maternity/postnatal care, changes to the antenatal modules in GP trainee program | **Service Provision**  Delivering in-person training programs, online webinars, pre-recorded trainings, revising the RACGP Check modules | **1** | N | N | N |
|  |  |  | **2** | Y | Y | Y |
|  |  |  | **3** | N | N | N |
|  |  |  | **4** | Y | Y | Y |

GP: general practitioner. HDP: hypertensive disorders of pregnancy. CVD: cardiovascular disease. RACGP: Royal Australian College of General Practitioners.

# Additional File 2: Research team’s preferred intervention ideas

| **Investigative team member** | **Intervention idea** | | |
| --- | --- | --- | --- |
|  | **Number 1 ranking (most preferred)** | **Number 2 ranking (second choice)** | **Number 3 ranking (third choice)** |
| **1** | 5 | 7 | 8 |
| **2** | 8 | 5 | 7 |
| **3** | 2 | 8 | 7 |
| **4** | 2 | 5 | 7 |

# Additional File 3: SWOT analyses results from end-users

**SWOT analysis Intervention 1 to address the barrier:** In GP practices, there are insufficient resources and training enabling them to assess and manage lifestyle risk factors among women following hypertensive pregnancies.

| Develop a suite of resources on hypertensive pregnancies and heart disease risk, for GPs to use in consultation with and send to women that have had hypertensive pregnancies during their postnatal checks. | |
| --- | --- |
| **SWOT Criteria** | **Summary of results** |
| **Strengths** | - Accessible for both GPs and women with a history of HDP - An opportunity to provide consistent care and resources. - Encourages women to proactively attend periodic monitoring. |
| **Weaknesses** | - Still relies on patient disclosing and/or GP knowing about pregnancy history. - Awareness and utilisation of the resources - Overwhelm of information - Lack of time during consult |
| **Opportunities**  How is this intervention better than the current model of care? | - Better consistency of care to women with a history of HDP - Allows women to take their health care into their own hands. - Better opportunity for education for both women with a history of HDP and GPs |
| **Threats**  What factors will lessen the impact of this intervention? | - GP awareness of the resources (requires some kind of training/in-service to make them aware) - Resources need to come with communication and not just handed out - Resources need to be widely available and always have up-to-date information |

GP: general practitioner

**SWOT analysis Intervention 2 to address the barrier:** GPs lack the confidence and skills to provide heart disease risk assessment and management with women after hypertensive pregnancies.

| Arrange for the Hunter New England Health Pathways postnatal check and hypertensive pregnancy modules to be updated to include information about heart disease prevention after hypertensive pregnancies. | |
| --- | --- |
| **SWOT Criteria** | **Summary of results** |
| **Strengths** | - Better consistency of care and a ‘gold standard of care’ - Accessible for GPs in HNE - Education plus reinforcement of existing knowledge |
| **Weaknesses** | - Use of, and awareness of HealthPathways - Requires training/in-service to make GPs aware of the updates. - Time constraints - Costs involved with making these changes |
| **Opportunities**  How is this intervention better than the current model of care? | - Consistency of care - Equal opportunity for GPs to upskill and access information. - Encourages GPs to ask all women about their pregnancy history |
| **Threats**  What factors will lessen the impact of this intervention? | - Needs to be written with all of HNE in mind (e.g., rural/remote/regional) - GPs need to be aware of this platform - Reliance on patients attending their appointments |

GP: general practitioner. HNE: Hunter New England

**SWOT analysis intervention 3 to address the barrier:** GPs are not being informed of their patients’ obstetric histories, including occurrence of hypertensive pregnancies from hospital discharge summaries.

| Information about hypertensive pregnancies and heart disease risk to be added into the discharge summary bundle sent home with women for themselves and their GP. | |
| --- | --- |
| **SWOT Criteria** | **Summary of results** |
| **Strengths** | - Closes the loop in communication between hospitals and primary care. - Allows women to take ownership of their health care. - Does not rely on patients remembering information verbally delivered. |
| **Weaknesses** | - Relies on GPs receiving the discharge summary/women attending GP appointments. - Relies on hospital staff adding information into these summaries. - GP and/or women may not read the summary/might miss something. |
| **Opportunities**  How is this intervention better than the current model of care? | - Enables GPs to receive a more accurate picture of women’s pregnancy history. - More effective transfer of communication between hospitals and primary care. - Acts as a prompt for review. |
| **Threats**  What factors will lessen the impact of this intervention? | - Patients might not bring or receive the discharge summary. - Time constraints of GPs (having to look at all the information in the bundle) - Time constraints of hospital staff in implementing this. |

GP: general practitioner.
