# Supplementary material for: Co-designing an intervention for cardiovascular disease risk assessment and management after hypertensive disorders of pregnancy in primary care
Source: Health Res Policy Syst. 2025 Feb 20;23:23. doi: 10.1186/s12961-024-01269-6 (PMC11844034; doi:10.1186/s12961-024-01269-6)
Supplement: Supplementary file 2 — Additional file 2. [file 12961_2024_1269_MOESM2_ESM.docx]

# Additional File 2: Research team’s preferred intervention ideas

| **Investigative team member** | **Intervention idea** | | |
| --- | --- | --- | --- |
|  | **Number 1 ranking (most preferred)** | **Number 2 ranking (second choice)** | **Number 3 ranking (third choice)** |
| **1** | 5 | 7 | 8 |
| **2** | 8 | 5 | 7 |
| **3** | 2 | 8 | 7 |
| **4** | 2 | 5 | 7 |
