# Supplementary material for: Co-designing an intervention for cardiovascular disease risk assessment and management after hypertensive disorders of pregnancy in primary care
Source: Health Res Policy Syst. 2025 Feb 20;23:23. doi: 10.1186/s12961-024-01269-6 (PMC11844034; doi:10.1186/s12961-024-01269-6)
Supplement: Supplementary file 3 — Additional file 3. [file 12961_2024_1269_MOESM3_ESM.docx]

# Additional File 3: SWOT analyses results from end-users

**SWOT analysis Intervention 1 to address the barrier:** In GP practices, there are insufficient resources and training enabling them to assess and manage lifestyle risk factors among women following hypertensive pregnancies.

| Develop a suite of resources on hypertensive pregnancies and heart disease risk, for GPs to use in consultation with and send to women that have had hypertensive pregnancies during their postnatal checks. | |
| --- | --- |
| **SWOT Criteria** | **Summary of results** |
| **Strengths** | - Accessible for both GPs and women with a history of HDP - An opportunity to provide consistent care and resources. - Encourages women to proactively attend periodic monitoring. |
| **Weaknesses** | - Still relies on patient disclosing and/or GP knowing about pregnancy history. - Awareness and utilisation of the resources - Overwhelm of information - Lack of time during consult |
| **Opportunities**  How is this intervention better than the current model of care? | - Better consistency of care to women with a history of HDP - Allows women to take their health care into their own hands. - Better opportunity for education for both women with a history of HDP and GPs |
| **Threats**  What factors will lessen the impact of this intervention? | - GP awareness of the resources (requires some kind of training/in-service to make them aware) - Resources need to come with communication and not just handed out - Resources need to be widely available and always have up-to-date information |

GP: general practitioner

**SWOT analysis Intervention 2 to address the barrier:** GPs lack the confidence and skills to provide heart disease risk assessment and management with women after hypertensive pregnancies.

| Arrange for the Hunter New England Health Pathways postnatal check and hypertensive pregnancy modules to be updated to include information about heart disease prevention after hypertensive pregnancies. | |
| --- | --- |
| **SWOT Criteria** | **Summary of results** |
| **Strengths** | - Better consistency of care and a ‘gold standard of care’ - Accessible for GPs in HNE - Education plus reinforcement of existing knowledge |
| **Weaknesses** | - Use of, and awareness of HealthPathways - Requires training/in-service to make GPs aware of the updates. - Time constraints - Costs involved with making these changes |
| **Opportunities**  How is this intervention better than the current model of care? | - Consistency of care - Equal opportunity for GPs to upskill and access information. - Encourages GPs to ask all women about their pregnancy history |
| **Threats**  What factors will lessen the impact of this intervention? | - Needs to be written with all of HNE in mind (e.g., rural/remote/regional) - GPs need to be aware of this platform - Reliance on patients attending their appointments |

GP: general practitioner. HNE: Hunter New England

**SWOT analysis intervention 3 to address the barrier:** GPs are not being informed of their patients’ obstetric histories, including occurrence of hypertensive pregnancies from hospital discharge summaries.

| Information about hypertensive pregnancies and heart disease risk to be added into the discharge summary bundle sent home with women for themselves and their GP. | |
| --- | --- |
| **SWOT Criteria** | **Summary of results** |
| **Strengths** | - Closes the loop in communication between hospitals and primary care. - Allows women to take ownership of their health care. - Does not rely on patients remembering information verbally delivered. |
| **Weaknesses** | - Relies on GPs receiving the discharge summary/women attending GP appointments. - Relies on hospital staff adding information into these summaries. - GP and/or women may not read the summary/might miss something. |
| **Opportunities**  How is this intervention better than the current model of care? | - Enables GPs to receive a more accurate picture of women’s pregnancy history. - More effective transfer of communication between hospitals and primary care. - Acts as a prompt for review. |
| **Threats**  What factors will lessen the impact of this intervention? | - Patients might not bring or receive the discharge summary. - Time constraints of GPs (having to look at all the information in the bundle) - Time constraints of hospital staff in implementing this. |

GP: general practitioner.
